# A Potential Mechanism of Tumor Progression during Systemic Infections Via the Hepatocyte Growth Factor (HGF)/c-Met Signaling Pathway

**DOI:** 10.3390/jcm9072074

**Published:** 2020-07-01

**Authors:** Hironori Tsujimoto, Hiroyuki Horiguchi, Yusuke Matsumoto, Risa Takahata, Nariyoshi Shinomiya, Takao Yamori, Hiromi Miyazaki, Satoshi Ono, Daizoh Saitoh, Yoji Kishi, Hideki Ueno

**Affiliations:** 1 Department of Surgery, National Defense Medical College, 3-2 Namiki, Tokorozawa 359-8513, Japan; savina_tomash@hotmail.com (H.H.); myusuke-st@ezweb.ne.jp (Y.M.); rtakahata@ndmc.ac.jp (R.T.); sono@shinkuki-hp.jp (S.O.); ykishi-3su@ndmc.ac.jp (Y.K.); ueno_surg1@ndmc.ac.jp (H.U.); 2 Department of Integrative Physiology and Bio-Nano Medicine, National Defense Medical College, 3-2 Namiki, Tokorozawa 359-8513, Japan; shinomi@ndmc.ac.jp; 3 Pharmaceuticals and Medical Devices Agency, 3-3-2 Kasumigaseki, Chiyoda-ku, Tokyo 100-0013, Japan; yamori-takao@pmda.go.jp; 4 Division of Traumatology, National Defense Medical College Research Institute, 3-2 Namiki, Tokorozawa 359-8513, Japan; hiromyzk@ndmc.ac.jp (H.M.); ds0711@ndmc.ac.jp (D.S.)

**Keywords:** liver metastasis, postoperative infectious complications, prognosis, HGF/c-Met signal pathway

## Abstract

Background: Increasing evidence has demonstrated that postoperative infectious complications (PICs) after digestive surgery are significantly associated with negative long-term outcomes; however, precise mechanisms of how PICs affect the poor long-term survival remain unclear. Here, we focused on the hepatocyte growth factor (HGF)/c-Met signaling pathway as one of those mechanisms. ***Methods****:* In the clinical setting, serum HGF levels were measured in the patients with sepsis and those with PICs after undergoing esophagectomy. Using a liver metastasis mouse model with cecal ligation and puncture (CLP), expressions of HGF and the roles of the HGF/c-Met pathway in the progression of tumor cells were examined. ***Results****:* Serum HGF levels were very high in the patients with intra-abdominal infection on postoperative days (PODs) 1, 3, and 5; similarly, compared to the patients without PICs, those with PICs had significantly higher serum HGF levels on 1, 3, and 5 days after esophagectomy. The patients with PICs showed poorer overall survival than those without PICs, and the patients with high serum HGF levels on POD 3 showed poorer prognosis than those with low HGF levels. Similarly, at 24 and 72 h after operation, serum levels of HGF in CLP mice were significantly higher than those in sham-operated mice. Intraperitoneal injection of mouse recombinant HGF significantly promoted liver metastases in sham-operated mice on 14 days after surgery. Knocking down c-Met expression on NL17 tumor cells by RNAi technology significantly inhibited the promotion of CLP-induced liver metastases. ***Conclusions****:* Infections after surgery increased serum HGF levels in the clinical as well as experimental settings. Induction of high serum HGF levels by CLP promoted liver metastases in a murine liver metastasis model, suggesting the involvement of the HGF/c-Met signaling pathway in tumor promotion mechanisms. Thus, targeting the HGF/c-Met signaling pathway may be a promising approach for malignant tumors, particularly in the patients with PICs.

## 1. Introduction

In recent years, postoperative infectious complications (PICs) of malignant tumors have been found to be associated with unfavorable long-term outcomes in various malignancies [1,2,3]. However, there is no clear consensus regarding the mechanisms underlying the association between the development of PICs and adverse prognostic outcomes [4].

Oncogene Met, which encodes a receptor-type tyrosine kinase, is a master signaling molecule that regulates the proliferation of tumor cells in the primary sites, invasion, and metastasis in surrounding tissues or remote sites, angiogenesis, peritoneal dissemination, and proliferation in metastatic lesions [5,6,7,8,9]. In 1991, Bottaro et al. and Naldini et al. demonstrated that hepatocyte growth factor (HGF) is a potential ligand for the Met receptor [10,11]. Since then, data showing that the HGF/c-Met signaling pathway might contribute to the progression of tumors have been increasing [12,13].

HGF is a potent mitogen for hepatocyte primary cultures [14] and stimulates the growth of various epithelial cells, such as renal tubular epithelial cells, keratinocytes, endothelial cells, and melanocytes [11]. In addition, HGF is substantially produced during surgical trauma and infectious stress such as sepsis, and plays an important role as a powerful mediator to induce protective effects against damaged tissues after surgical stress [15,16,17,18].

We have previously reported that persistent abdominal infection promotes liver metastases in a murine liver metastasis model [9]. Thus, we hypothesized that enhanced HGF production caused by infection may contribute to tumor progression via the activation of the HGF/c-Met signaling pathway. To validate this notion, we measured the serum HGF levels in the patients with sepsis and those with PICs after esophagectomy for esophageal cancer in the clinical settings. We also employed a previously described animal model [9] to investigate the role of HGF/c-Met signaling in liver metastasis.

## 2. Experimental Section

### 2.1. Materials/Patients and Methods

#### 2.1.1. Clinical Study

A total of 16 patients with emergent laparotomy for an intra-abdominal infection and 118 consecutive patients who underwent esophagectomy for esophageal cancer at the National Defense Medical College Hospital were included in this study. Blood samples were collected from the patients who underwent esophagectomy as the following time schedules; immediately after induction of anesthesia (within 5 min); immediately after surgery (within 30 min); and on postoperative days (PODs) 1, 3, and 5. Blood samples were also collected from the patients with sepsis immediately after admission, immediately after surgery (within 30 min after surgery), and on PODs 1, 3, 5, and 7. All samples were collected with non-pyrogenic, sterile Falcon tubes. Then blood samples were subjected to cold centrifugation at 1500× *g* for 10 min and the obtained serum was stored at −80 °C. To improve the homogeneity of measurements, all samples were simultaneously analyzed with the same assay reagents by the same laboratory technicians. Serum cytokine levels were measured using enzyme-linked immunosorbent assay kits (human IL-6: BD Biosciences, MD, USA; HGF: R&D Systems, MN, USA).

To assess the severity of sepsis, the patients were evaluated with the Acute Physiology and Chronic Health Evaluation (APACHE) II scoring system [19] and the duration that met the criteria of systemic inflammatory response syndrome [20]. Clinical stages of the patients with esophageal cancer were evaluated using the International Union Against Cancer’s TNM Classification of Malignant Tumors [21]. In the current study, we compared the overall survival between the patients with and without PICs, and between the patients whose serum HGF levels on POD 3 were three times or more than the preoperative values and those less than three times, because the median of the POD 3/preoperative HGF levels was 2.90.

#### 2.1.2. Animal Study

##### Mice and Cell Line

Female (8–10 weeks old) BALB/c mice were purchased from Japan SLC (Hamamatsu, Japan), and were provided with food and water ad libitum. NL-17, a murine colon cancer cell line derived from colon 26 cells, possessing high ability of potent liver metastasis, was a kind gift by the Division of Molecular Pharmacology, Cancer Chemotherapy Center (Japanese Foundation for Cancer Research, Tokyo, Japan). The cells were maintained in Roswell Park Memorial Institute (RPMI) 1640 medium containing 5% heat-inactivated fetal bovine serum and antibiotics in an atmosphere of 5% CO_2_ at 37 °C.

##### Animal Model

Polymicrobial peritonitis was induced in the murine model by cecal ligation and puncture (CLP), as previously described [22]. Briefly, after anesthetization with an intraperitoneal injection of pentobarbital, the anterior abdominal wall of the mice was shaved, a small incision was made to expose the cecum, and it was ligated at its base with 3-0 silk. To induce peritonitis, the ligated portion was punctured once with a 23-gauge needle. Next, the cecum was returned to the abdominal cavity, and 0.5 mL normal saline was administered intraperitoneally before closing the abdomen. Similar surgical stress was induced in uninfected controls via sham surgery (i.e., the same procedure without CLP; sham-treated mice). Preliminary experiments demonstrated that the mortality rates of the peritonitis model (CLP mice) were < 25% at 14 days after surgery.

To induce liver metastasis in the experimental murine model, a left subcostal incision without laparotomy was immediately made after CLP or sham operation or the administration of mouse recombinant HGF or normal saline, and 1 × 10^5^ NL-17 cells suspended in 100 μL of Hanks balanced salt solution containing 1% BALB/c murine serum were inoculated into the spleen with a 27-gauge needle via the peritoneum [9]. Day 0 was defined as the day of inoculation into the spleen.

To evaluate the direct effect of HGF on liver metastasis, 5 μg/100 μL recombinant mouse HGF (R&D Systems, Inc., Minneapolis, MN, the USA) or 100 μL of normal saline was intraperitoneally administered, and liver metastases were assessed 14 days after the injections.

##### Flow Cytometric Analysis of c-Met Expression

NL-17 cells (5 × 10^5^ cells/100 μL) were incubated with FITC conjugated anti-mouse c-Met antibody (Thermo Fisher Scientific, Tokyo, Japan) or its isotype control (anti-mouse IgG1, kappa, Thermo Fisher Scientific) for 15 min at 4 °C. The samples were analyzed using the FC500 cytometer (Beckman Coulter, Pasadena, CA, USA). Flow cytometric analysis revealed that 85% of NL-17 cells expressed c-Met (Figure S1).

##### Measurement of Cytokines

The serum cytokine levels (interleukin (IL)-6, IL-10, and HGF) at pretreatment condition and at 12, 24, and 72 h after CLP or sham operation were measured using the enzyme-linked immunosorbent assay kits (IL-6 and IL-10: BD Biosciences, San Jose, CA, the USA; HGF: R&D Systems, Inc.).

##### Establishment of c-Met Knockdown Cells

c-Met knockdown cells were established using the RNA interference technology, as previously described [23]. Briefly, NL-17 cells were infected with adenovirus (clone 178M) containing siRNA encoding against murine c-Met at 100 multiplicity of infection (moi) and were incubated for 72 h in an atmosphere of 5% CO_2_ at 37 °C. c-Met knockdown was confirmed by measuring the expression level of c-Met using Western blotting. NL-17 cells infected with nonspecific adenovirus (clone GL-2 that encodes siRNA against firefly luciferase) under the same condition were used as a control.

##### Western Blotting

Protein was extracted from NL-17 cells by treatment with Complete Lysis Buffer (50 mM HEPES (pH7.5), 5 mM EDTA, 50 mM NaCl, 10 mM NaPPi, 50 mM NaF, 1% NP-40, 0.01 M Na3VO4) containing protease inhibitor cocktails (cOmplete™ Protease Inhibitor Cocktail, Roche) on ice for 15 min, followed by centrifugation at 15,000 rpm for 10 min. The supernatant was stored at −80 °C until use for Western blotting. After boiling with sample buffer in the reduced condition (Tris-HCl (pH 6.8) 40 mM, SDS 1%, Glycerol 5%, Bromophenol blue 0.0003%, DTT 0.05 M), the cell extracts were separated using SDS-PAGE and were transferred to polyvinylidene difluoride membranes (Invitrogen). The membranes were incubated with antibodies against Met (SP260: sc-162, Santa Cruz Biotechnology, Santa Cruz, CA, USA) or β-actin (AC-15: ab6276, Abcam, Cambridge, MA, USA) followed by horseradish peroxidase-conjugated secondary antibodies (Santa Cruz Biotechnology). After incubation with ECL reagent (Amersham Biosciences, Buckinghamshire, United Kingdom), chemiluminescence signals were photographed and quantitated via image analysis.

Western blotting analysis confirmed that c-Met expression was effectively inhibited in NL-17 cells infected with 178M in a moi-dependent manner (Figure S2).

##### Evaluation of Tumor Growth in Liver Metastasis

The extent of liver metastasis was evaluated using liver weight because our previous study revealed that liver weight significantly correlated with the extent of liver metastasis, which was determined based on evaluation using liver tissue sections [9]. To assess the potential of tumor growth at the liver metastatic site, immunohistochemical staining of mini-chromosome maintenance protein 7 (MCM7) was performed. In brief, the specimens were fixed with 10% formalin and embedded in paraffin wax. Then, the samples were sectioned into 4-µm-thick slices. Next, after deparaffinization with xylene, the sections were autoclaved for antigen retrieval. The activity of endogenous peroxidase was quenched using 5% H_2_O_2_. The sections were incubated in 4% skimmed milk to block nonspecific binding and in MCM7 monoclonal antibody, which is the primary antibody, overnight at 4 °C (DCS-141, Santa Cruz Biotechnology). Subsequently, the sections were incubated with secondary antibody (EnVision+System HRP Labelled Polymer Anti-mouse, DAKO, Tokyo, Japan) for 1 h at room temperature and in 0.1% diaminobenzidine tetrahydrochloride for 5 min and were counterstained with hematoxylin for antigen visualization. The largest sections obtained from three different liver lobes were examined and independently evaluated in a blinded fashion by two surgeons who were familiar with surgical pathology. The labeling index of MCM7 was scored with sections visualized at a magnification of 400× as the ratio of MCM7 positive cells/tumor cells.

##### Ethics

Informed consent for clinical study was obtained from all individuals or their family before the initiation of this study. The protocol was based on the ethical guidelines of the 2013 Declaration of Helsinki and was reviewed and approved by the Institutional Ethics Committee of the National Defense Medical College (Saitama, Japan) (Approval number 3049). All animal protocols were based on the Animal Research: Reporting of In Vivo Experiments (ARRIVE) guidelines and were approved by the Institutional Review Board for the Care of Animal Subjects of the National Defense Medical College (Approval number 10073).

##### Statistical Analysis

Statistical analyses were performed using JMP Pro 14.0.0 (SAS Institute, Cary, NC, USA). Data were expressed as mean ± SEM. Statistical methods used in this study were Mann–Whitney U test or chi-square test with Fisher’s exact test, as appropriate. Survival rates were calculated using the Kaplan–Meier method, and the significance of the differences in survival rates was determined using the log-rank test. *p*-values of < 0.05 were considered statistically significant.

## 3. Results

### 3.1. Clinical Study

Demographic data of the patients with sepsis and esophageal cancer are depicted in Table 1 and Table 2, respectively. In the patients with sepsis, the most frequent cause was colonic perforation, followed by gangrene in the intestine owing to superior mesenteric artery occlusion. The mean APACHE II score was 11.7 ± 3.4, and the 30-day mortality rate was 19% (Table 1).

Looking at the patients who underwent esophagectomy, the presence of PIC was not associated with factors such as age, sex, administration of neoadjuvant therapy, tumor depth, nodal involvement, and pathological stages (Table 2).

The patients with PICs had significantly higher rates of tumors located in the upper thorax than those without PICs. In addition, 3-field lymphadenectomy was more frequently performed in the patients with PICs. Anastomotic leakage was the most frequent infectious complication, followed by pneumonia.

**Figure 1:**
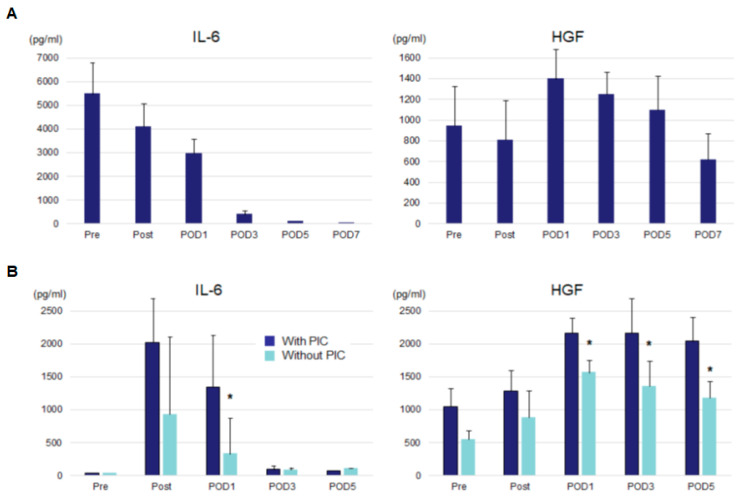
Serum IL-6 and HGF levels of patients with sepsis (**A**) and those who underwent esophagectomy (**B**). IL-6, interleukin-6; HGF: hepatocyte growth factor, POD: postoperative day, PICs: postoperative infectious complications. * *p* < 0.05 vs. patients with PIC.

Serum IL-6 and HGF levels in the patients with sepsis and esophageal cancer are depicted in Figure 1. The patients with sepsis had extremely elevated serum IL-6 levels at admission (Pre), which gradually decreased until POD 3 (Figure 1A). Conversely, serum HGF levels peaked at POD 1 and showed increased levels until POD 5. Serum IL-6 levels of the patients who underwent esophagectomy were high immediately after surgery until POD 1. The patients with PICs showed significantly higher serum IL-6 levels on POD 1 than those without PICs (Figure 1B). Serum HGF levels increased on POD 1 as compared with preoperative values and remained high until POD 5. Again, the patients with PICs had significantly higher serum HGF levels than those without PICs, showing statistical significance on PODs 1, 3, and 5 (*p* < 0.05).

**Figure 2:**
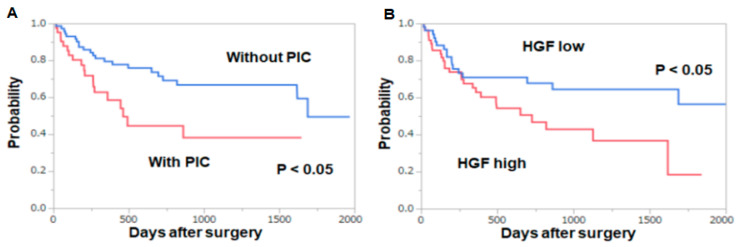
Overall survival after esophagectomy. (**A**) Comparison between the patients with and without PICs (**B**) Comparison between the patients whose HGF levels on POD 3 were three times or more higher than the preoperative values and of those less than three times higher. Survival rates were calculated using the Kaplan–Meier method, and the significance of the difference in survival rates was determined using the log-rank test.

The patients with PICs had poorer overall survival than those without PICs (*p* < 0.05, Figure 2A). Similarly, the patients whose HGF levels on POD 3 increased three times or more than the preoperative values showed poorer overall survival than those showing less than three times changes (*p* < 0.05, Figure 2B).

### 3.2. Animal Study

**Figure 3:**
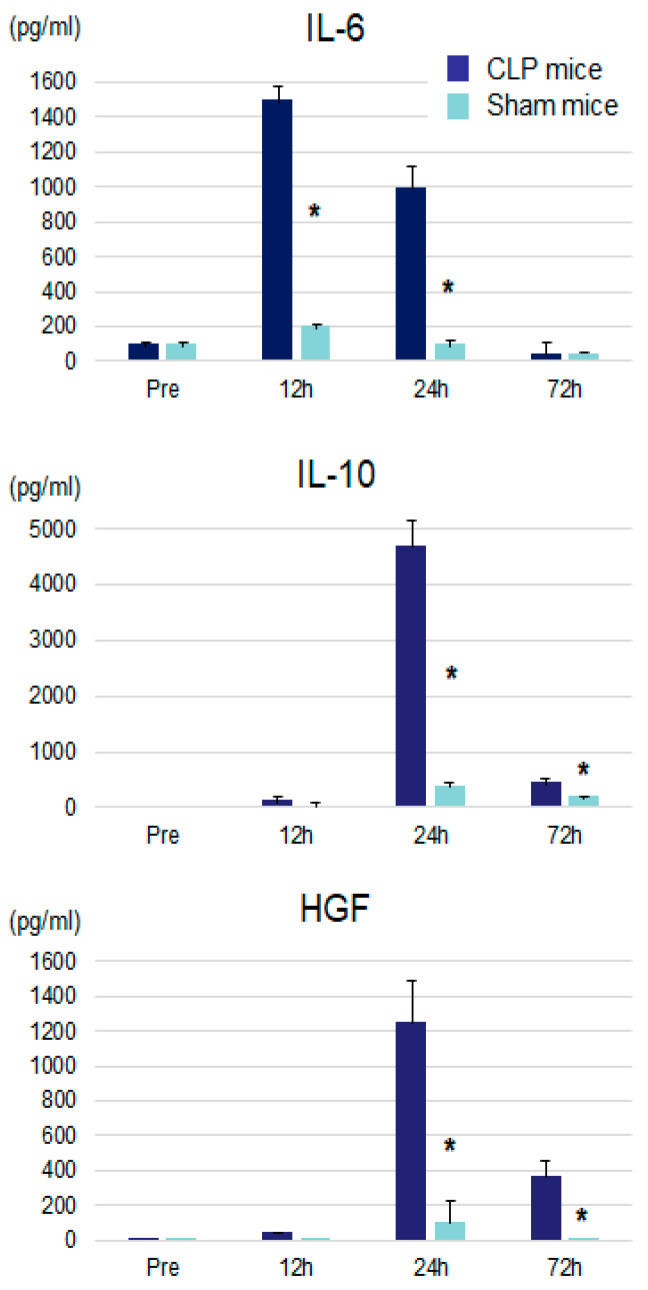
Serum IL-6, IL-10, and HGF levels after CLP or sham operation. IL: interleukin, HGF: hepatocyte growth factor, CLP: cecal ligation and puncture. * *p* < 0.05 vs. CLP mice.

Serum IL-6 levels of CLP mice peaked at 12 h after surgery and were significantly higher than those of sham-treated mice at 12 and 24 h after surgery (Figure 3). Serum IL-10 and HGF levels peaked at 24 h after surgery and were significantly higher at 24 and 72 h after surgery as compared with those of sham-treated mice. These cytokine changes suggest CLP provided inflammatory conditions and affected the growth of implanted tumor cells.

**Figure 4:**
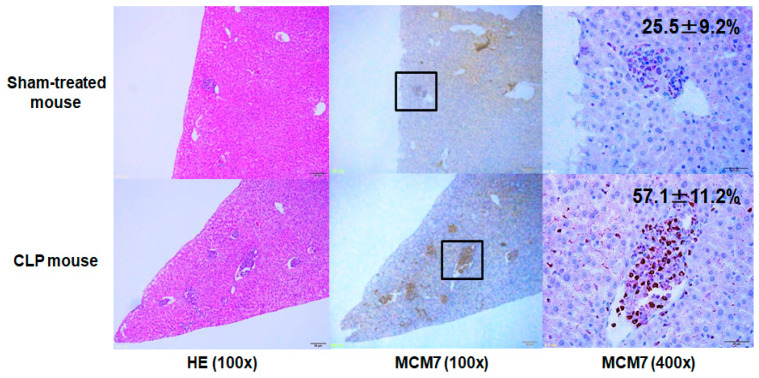
Representative images of HE and MCM7 staining of liver metastases at 7 days after surgery in sham-treated and CLP mice. The mean MCM7 labeling indices were inserted in each right upper panel. At least five independent experiments were performed in both sham-treated and CLP mice, and similar results were obtained.

Representative images of liver metastases with MCM7 staining at 7 days after surgery in sham-treated and CLP mice are depicted in Figure 4. Metastatic lesions of CLP mice had a consistently higher labeling index of MCM7 than that of sham-treated mice (57.1 ± 11.2% vs. 25.5 ± 9.2%), indicating that an intra-abdominal infection caused by CLP enhanced tumor cell proliferation in the metastatic sites.

**Figure 5:**
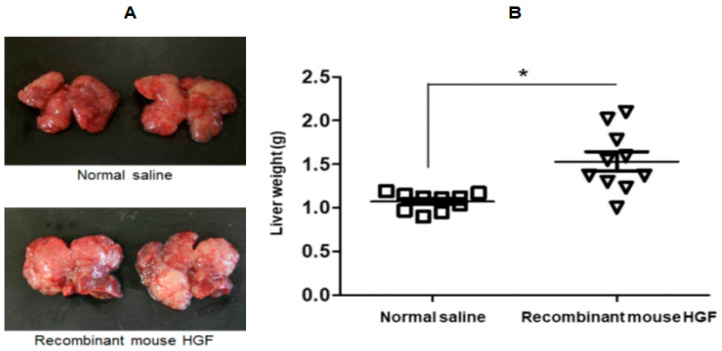
Intraperitoneal injection of recombinant mouse HGF significantly promoted liver metastases. (**A**): Representative image of macroscopic liver metastases on day 14 are depicted. (**B**): The liver weights 14 days after intraperitoneal injection of recombinant mouse HGF (*n* = 10) or normal saline (*n* = 10) in the murine liver metastasis model.

Next, we investigated if HGF had a direct effect on liver metastasis in sham-treated mice. On day 14, liver metastatic nodules showed remarkable growth in mice that were administered with recombinant mouse HGF (Figure 5A). Compared with the injection of normal saline, intraperitoneal injection of recombinant mouse HGF significantly promoted liver metastases at day 14 after surgery as assessed by the liver weight (Figure 5B).

**Figure 6:**
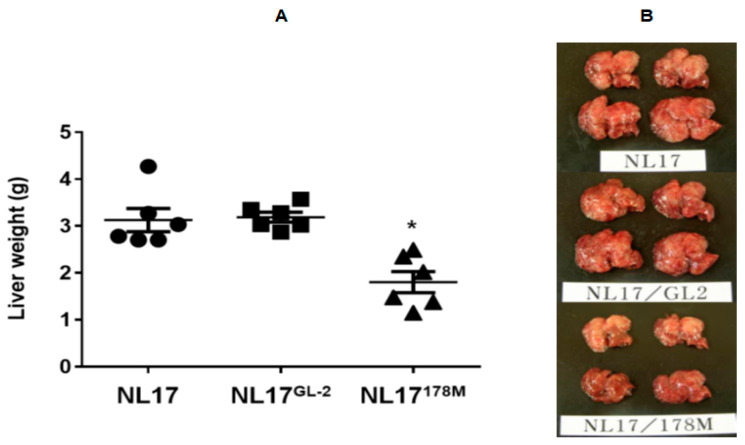
The liver weights 14 days after CLP using NL-17 cells with 178M and control virus in the murine liver metastasis model. A: Liver metastasis of NL17 cells infected with control virus (NL17^GL-2^) showed similar levels to that of noninfected NL17 cells at 14 day after CLP. In contrast, significant decrease in liver metastasis was observed in NL17 cells infected with 178M virus at moi 100 (NL17^178M^) (*n* = 6). * *p* < 0.05 vs. noninfected NL17 cells and NL17 cells infected with control virus (NL17^GL-2^). B: Representative images of macroscopic liver metastases on day 14 are depicted.

The group of NL-17 cells infected with control virus (NL-17^GL-2^) showed a similar liver metastasis level to that of uninfected NL-17 cells in CLP mice at 14 days after surgery, whereas a significant decrease in liver metastasis was observed in the group of NL-17 cells infected with 178M (NL-17^178M^) that targeted the HGF receptor, c-Met at 100 moi (Figure 6).

## 4. Discussion

This study first showed that patients with sepsis and PICs after esophagectomy had elevated serum HGF levels in the clinical setting. Moreover, the HGF/c-Met signaling pathway directly contributed to the promotion of liver metastasis during sepsis in the animal model.

We have previously demonstrated that an intra-abdominal infection induced by CLP promoted liver metastasis resulting in poor cancer-related survival [9]. The process of tumor metastasis after injecting tumor cells into the spleen can include the promotion of release of tumor cells in the spleen, penetration into the vessels of tumor cells, adhesion to the hepatic tissue, and acceleration of tumor cell proliferation in the metastatic focus [24]. Thus, various mechanisms during infection leading to a poor prognosis have been involved in each process. However, the main mechanism in each step remains unknown.

Disseminated intestinal bacteria may directly influence the innate immune system in the abdominal cavity, or sepsis may induce local and systemic immune responses with different cytokine productions, which may cause tumor proliferation [25]. In this study, we demonstrated that the administration of recombinant mouse HGF promoted liver metastases in sham mice, suggesting that the HGF/c-Met signaling pathway is at least in part involved in the promotion of liver metastasis.

*Helicobacter pylori* (HP) and its lipopolysaccharide (LPS), which is a component of its outer membrane, significantly augmented gastric cancer cell growth via the LPS-Toll like receptor 4 pathway [26]. In addition, our previous report has shown that c-Met expression predicted poor prognosis for gastric cancers with HP-positive infection but not for those with HP-negative infection [27]. Persistent HP infection in the gastric mucosa may promote the production of cytokines, including HGF, in the microenvironment, which could be associated with tumor progression via stimulating c-Met signaling in gastric cancer cells. Thus, the hypothesis that systemic infection is involved in the tumor growth via the HGF/c-Met signaling pathway may be reasonable.

In this study, patients with sepsis and PIC had higher serum HGF levels in the clinical setting. Moreover, patients with PIC had poorer overall survival than those without PIC, which is consistent with the results of our previous reports [1]. Takahashi et al. demonstrated that patients with colorectal cancer who have high levels of HGF prior to treatment had shorter progression-free survival as well as overall survival compared with those with low levels of HGF [12]. Kubo et al. reported that serum HGF levels are correlated with tumor stage in melanoma patients [28]. Regarding the postoperative serum HGF levels, we showed that patients with high HGF levels on POD 3 had poorer overall survival than those with low HGF levels, suggesting that enhanced postoperative HGF levels are associated with tumor progression in esophageal cancer. Yang et al. demonstrated that serum HGF levels were significantly elevated at POD 3 compared with preoperative values in pancreatic cancer [29]; however, postoperative change in HGF levels was not correlated with overall survival as evaluated via a multivariate analysis. Thus, the association between higher postoperative HGF levels and prognosis may differ based on the tumor type (e.g., histological type and expression of c-Met).

Several in vivo studies have demonstrated that the activation of the HGF/c-Met signaling pathway causes cancer invasion and metastasis, which supports and broadens the results of this study [30,31]. In addition, several monoclonal antibodies that block c-Met (onartuzumab, emibetuzumab, LY3164530, etc.) or HGF (rilotuzumab, ficlatuzumab, YYB-101, etc.) have been used for current clinical trials [32]. However, most previous studies have demonstrated minimal or no significant clinical benefit, which was less than expected. One potential explanation for these disappointing results is that the effect of HGF produced by PIC is not always considered in these studies. Thus, the incidence of PIC, which is associated with enhanced serum HGF levels, may be a better biomarker for patient selection in future trials that assess the HGF/c-Met signaling pathway.

## 5. Conclusions

Persistent inflammation due to infection was shown to induce higher serum HGF levels in the clinical and experimental settings. In addition, using a murine liver metastasis model, it was suggested that higher systemic HGF levels can contribute to the promotion of liver metastases, which indicates that the HGF/c-Met signaling pathway may be a key mechanism involved. Thus, treatment of malignant tumors focusing on the HGF/c-Met signaling pathway may be a promising strategy, particularly for the patients with PICs.

## Figures and Tables

**Table 1:** Demographic data of patients with sepsis.

				Patients	
				(*n* = 16)	
Age				71.0 ± 9.4	
Male/Female				9/7	
Diagnosis					
	Colonic perforation			14	88%
	SMAO			2	13%
APACH II score				11.7 ± 3.4	
Duration of SIRS (days)				3.4 ± 2.4	
Length of stay in hospital (days)				27.4 ± 23.8	
Duration of SIRS (days)				1.8 ± 2.6	
30-day mortality				3	19%

SMAO: superior mesenteric artery occlusion, APACHE: acute physiology and chronic health evaluation, SIRS: systemic inflammatory response syndrome.

**Table 2:** Demographic data in patients with and without postoperative infectious complications.

		With PIC		Without PIC		*p*-Value
		(*n* = 44)		(*n* = 74)		
Age		70.9 ± 8.7		71.4 ± 8.6		0.48
Male/Female		38/6		60/14		0.46
Tumor location						
	Upper	10	23%	4	5%	0.01
	Middle	20	45%	33	45%	
	Lower	14	32%	37	50%	
Fields of lymphadenectomy						
	2-field	22	50%	53	72%	0.02
	3-field	22	50%	21	28%	
Neoadjuvant therapy						
	No	25	57%	31	42%	0.12
	Yes	19	43%	43	58%	
	Chemotherapy	19		42		
	Chemoradiation	0		1		
Tumor depth						
	pT1-pT2	17	39%	34	46%	0.44
	pT3-pT4	27	61%	40	54%	
Nodal metastasis						
	pN0-N1	35	80%	53	72%	0.34
	pN2-N3	9	20%	21	28%	
pStage						
	1	12	27%	22	30%	0.23
	2	10	23%	15	20%	
	3	18	41%	36	49%	
	4	4	9%	1	1%	
Infectious complications *						
	Anastomotic leakage	23	52%	-		
	Pneumonia	14	32%	-		
	Empyema	4	9%	-		
	ARDS	2	5%	-		
	UTI	2	5%	-		
	Others	4	9%	-		

PIC: postoperative infectious complications, ARDS: acute respiratory distress syndrome, UTI; urinary tract infection. * Four patients had two or more infectious complications.

## Supplementary Materials

The following are available online at https://www.mdpi.com/2077-0383/9/7/2074/s1, Figure S1: A representative flow cytometric histogram plot of c-Met expression in NL17 cells., Figure S2: NL17 cells infected with 178M virus inhibited c-Met expression in an m.o.i.-dependent manner.
