# Supplementary material for: A Potential Mechanism of Tumor Progression during Systemic Infections Via the Hepatocyte Growth Factor (HGF)/c-Met Signaling Pathway
Source: J Clin Med. 2020 Jul 1;9(7):2074. doi: 10.3390/jcm9072074 (PMC7408644; doi:10.3390/jcm9072074)
Supplement: Supplementary file 1 [file jcm-09-02074-s001.pdf]

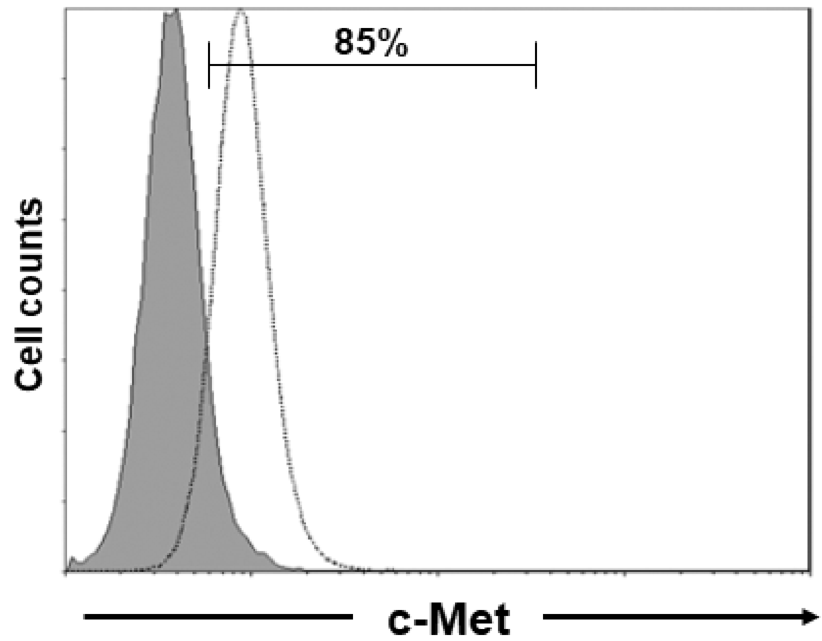

Supplementary Figure S1. A representative flow cytometric histogram plot of c-Met expression in NL17 cells. Filled histogram represents NL17 cells stained with isotype control antibody. The gray line represents NL17 cells stained with c-Met antibody. Approximately 85% of NL17 cells expressed c-Met.

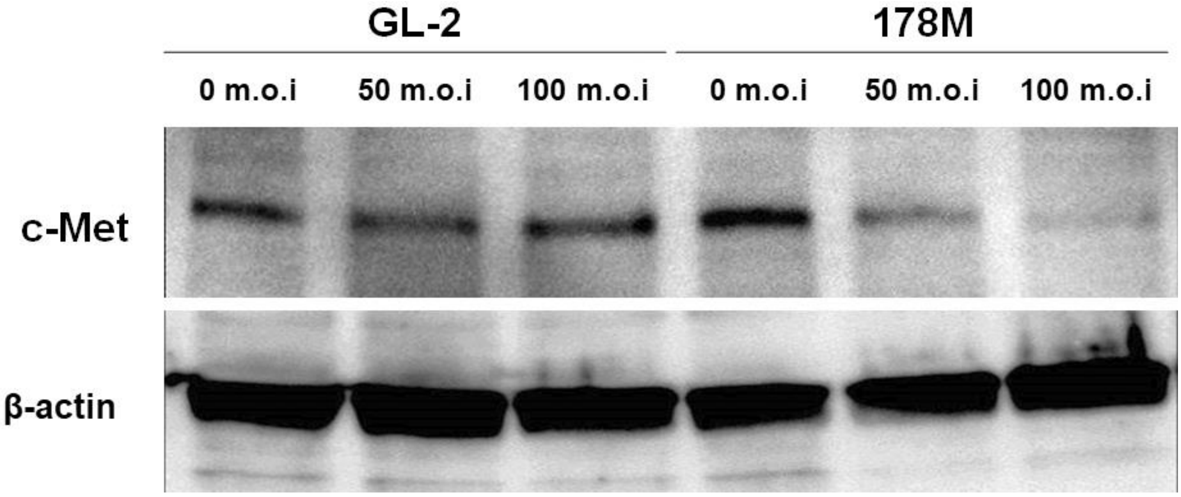

Supplementary Figure S2. NL17 cells infected with 178M virus inhibited c-Met expression in an m.o.i.-dependent manner ($NL17^{178M}$). Meanwhile, the NL17 cells infected with control virus ($NL17^{GL-2}$) did not inhibit c-Met expression. At least three independent experiments were performed, and similar results were obtained.
